# The Content and Location of 3-*O*-Sulfated Glucosamine Enable Differentiation of Ovine, Bovine and Porcine Heparins

**DOI:** 10.3390/biom16071025

**Published:** 2026-07-14

**Authors:** Arianna Somma, Elena Urso, Michela Parafioriti, Marco Guerrini, Antonella Bisio

**Affiliations:** Istituto di Ricerche Chimiche e Biochimiche G. Ronzoni, Via G. Colombo 81, 20133 Milan, Italy

**Keywords:** ovine, bovine, porcine heparins, AT-affinity chromatography, liquid-chromatography mass spectrometry, nuclear magnetic resonance spectroscopy

## Abstract

Heparin, the naturally occurring, highly sulfated glycosaminoglycan drug, acts as a regulator of diverse biological processes by interacting with diverse specific proteins through a variety of sulfated structural motifs with varying degrees of affinity. Despite its rarity, 3-*O*-sulfation of glucosamine plays a key role in several biological activities, especially anticoagulant activity mediated by interaction with antithrombin (AT), a mechanism that has been extensively studied. The present work primarily focuses on sequences containing 3-*O*-sulfated glucosamine that are not involved in anticoagulant activity, analysing their relative abundance and structural environments in heparins derived from distinct animal origins. Three heparin samples derived from bovine, ovine and porcine intestinal mucosa (BMH, OMH and PMH) were fractionated by affinity chromatography on AT-Sepharose into no affinity (NA) and high affinity (HA) fractions. Parent heparins and derived NA and HA fractions were enzymatically depolymerised using either a cocktail of heparinases I, II and III or heparinase II alone, and the resulting mixtures of di- and oligosaccharides were analysed by liquid chromatography coupled with mass spectrometry. Digestion with heparinase II, which preserves heparin sequences containing 3-*O*-sulfated glucosamine, produced a series of 3-*O*-sulfated trisaccharides containing two glucosamine residues either side of a uronic acid, located at the non-reducing end (NRE) of heparin chains. Treatment with a heparinase cocktail, which cleaves these trisaccharides, released NRE glucosamine monosaccharides, including 3-*O*-sulfated species. Notably, both the number of NRE trisaccharide species and the overall proportion of 3-*O*-sulfated monosaccharides were markedly higher in OMH compared to BMH or PMH. Using ^1^H/^13^C bi-dimensional nuclear magnetic resonance spectroscopy, the higher proportion of NRE 3-*O*-sulfated glucosamine in ovine heparin relative to bovine and porcine heparins was found to be preferentially located in chains lacking affinity for AT. These results offer new insights into the localisation of most 3-*O*-sulfated heparin sequences that are not associated with anticoagulant activity and, importantly, also reveal a possible structural marker that indicates the ovine origin of heparin. Additionally, a tetrasaccharide species was detected that may be indicative of a highly sulfated AT-binding site in BMH.

## 1. Introduction

Heparin, a life-saving animal-derived drug, entered clinical use approximately 90 years ago as a primary anticoagulant for the prevention and treatment of blood clots. Since then, particularly in its low-molecular-weight form, it has remained the most widely used anticoagulant for the treatment of thrombotic disorders and for hemodialysis [1]. The growing incidence of cardiovascular diseases, and consequently the implementation of ever-increasing endovascular procedures, is leading to a rising demand for heparin. This represents a critical issue because the heparin supply chain suffers from a dual vulnerability: reliance on a single animal source (porcine intestinal mucosa) and concentration within a single geographic region (China). The urgent need to diversify the source of heparin extraction is amplified by the risks of zoonoses, such as the lethal African Swine Fever, which can pose a significant threat to global heparin supplies [2].

The Food and Drug Administration (FDA) and other regulatory agencies have actively evaluated the reintroduction of bovine mucosal heparin (BMH) for several years. While BMH remains clinically available in several countries, such as Brazil and Argentina, alternative formulations like ovine mucosal heparin (OMH) have also recently been introduced in certain Asian countries [3,4]. Several studies have reported comparable efficacy, safety and pharmacologic profile for both BMH and OMH and porcine mucosal heparin (PMH) [5,6,7,8,9]. However, considering the well-documented highly complex nature of heparin, detailed and in-depth comparative studies are still needed to identify the potentially subtle structural differences that may influence their pharmacological activity.

Heparin, a highly sulfated glycosaminoglycan (GAG) composed of polymeric chains of varying length, consists of disaccharide units of α-d-glucosamine (A) linked 1,4 to α-L-iduronic acid 2-*O*-sulfate (I) or β-d-glucuronic acid (G), with variable substitution patterns, the most prevalent being 2-*O*-sulfated iduronic acid and *N*- and 6-*O*-sulfated glucosamine [10]. The predominant disaccharide motif in heparin is the trisulfated unit I_2S_–A_NS6S_, which forms the so-called *regular* regions of the polymer and accounts for more than 70% of the chain. The remaining ~30%, referred to as the *irregular* regions, displays a more heterogeneous composition that includes G and I2OH residues, *N*-acetylated glucosamine (A_NAc_) and the less abundant but functionally important 3-*O*-sulfated glucosamine residues, typically bearing additional *N*- and/or 6-*O*-sulfation. These 3-*O*-sulfated glucosamine residues are predominantly found within the specific pentasaccharide sequence A_NAc6S_–G–A_NS3S6S_–I_2S_–A_NS6S_, which binds with high affinity to antithrombin (AT) and thereby potentiates its anticoagulant activity [11,12]. Although the irregular regions do not appear to be distributed in an orderly manner, the 3-*O*-sulfated pentasaccharide turns out to be preferentially enriched toward the non-reducing termini of heparin chains [13,14,15,16]. Many studies conducted on heparan sulfate (HS), the GAG structurally analogous to heparin, have revealed that 3-*O*-sulfation, despite its rarity, is believed to play a critical role in various biological functions beyond anticoagulant activity, significantly contributing to the binding affinity of different saccharide sequences toward key proteins [17].

The present work is part of a larger research framework aiming to obtain new structural insights into the composition of heparin derived from intestinal mucosa of porcine, bovine and ovine origin, which could offer valuable information concerning both biosynthesis and extraction processes. Moreover, key parameters relevant to the quality control of the drug could also be highlighted. In this context, particular attention was paid to heparin sequences containing 3-*O*-sulfated glucosamine that do not interact with AT.

Three unfractionated heparin (UFH) samples derived from different animal source, i.e., BMH, OMH and PMH, were fractionated based on their affinity to antithrombin (AT) into no affinity (NA) and high affinity (HA) fractions through chromatography on AT-Sepharose. By employing enzymatic strategies with bacterial lyases that are specific and selective for particular heparin sequences, such as heparinase I, II and III in a cocktail and/or heparinase I and heparinase II alone, the NA and HA fractions and the UFHs were depolymerised. The resulting mixture of di- and oligo-saccharide products were analysed by liquid chromatography coupled with mass spectrometry (LC-MS). In particular, enzymatic digestion with heparinase II was employed to preserve the 3-*O*-sulfated glucosamine-containing sequences [18,19,20] and identify the residues adjacent to this important residue. The three parent UFHs and their resulting NA and HA fractions were also analysed using bi-dimensional nuclear magnetic resonance (2D-NMR) spectroscopy to evaluate their mono- and disaccharide composition as an orthogonal approach to LC-MS. Finally, two additional UFH samples per animal source were analysed by 2D-NMR and LC-MS following depolymerisation with the heparinase cocktail for comparative purpose.

The results obtained provide new and valuable insights into the location of most 3-*O*-sulfated sequences in heparin that are not involved in anticoagulant activity and also identify a possible structural marker indicative of the ovine origin of heparin.

## 2. Materials and Methods

### 2.1. Materials

Bovine mucosal heparins were provided by Prof. J. Fareed (Loyola University, Chicago, IL, USA), ovine mucosal heparins were obtained from Suzhou Ronnsi Pharma (Suzhou, China) and porcine mucosal heparins were obtained from Opocrin S.p.A. (Modena, Italy) and Hepalink (Shenzhen, China). All lyases, obtained from *Flavobacterium heparinum*, Heparinase I (EC 4.2.2.7), Heparinase II (no assigned EC number) and Heparinase III (EC 4.2.2.8) were from Grampian Enzymes (Aberdeen, Scotland, UK).

### 2.2. Heparin Fractionation by Affinity Chromatography on AT-Sepharose

The AT-Sepharose column (30 × 5 cm) was prepared according to the previously described procedure [21]. Preliminary experiments were conducted to determine the maximum binding capacity of the AT-Sepharose column by loading increasing amounts of a reference heparin sample. It was established that, for heparin loads of 120 mg or less, the relative percentages of the NA fractions remained constant. For each sample, 100 mg were dissolved in 10 mL of equilibrium buffer (0.4 M NaCl in 50 mM AcNH_4_ at pH 7.4) and loaded onto the column (flow rate: 10 mL/min). Heparin chains devoid of antithrombin affinity were subjected to elution with 1800 mL of equilibrium buffer, subsequently collected as a singular fraction designated as the NA fraction. Heparin chains exhibiting high affinity for antithrombin were eluted with 1600 mL of elution buffer (3 M NaCl in 50 mM AcNH_4_ at pH 7.4) and subsequently collected as a single fraction designated as the HA fraction. Subsequently, the column was washed with 500 mL of buffer composed of 3.5 M NaCl in 50 mM AcNH_4_ at pH 7.4. All the effluent fractions from AT-Sepharose column were analysed for uronic acid content by the carbazole reaction [22]. In each colorimetric assay, the calibration curve was constructed with the same sample solution loaded onto the AT-Sepharose column.

### 2.3. Desalting Procedure for Eluted Fractions

The NA fractions were initially desalted by dialysis using 3500 Da cut-off membranes and was completed by gel permeation chromatography using a HW 40S TSK resin with 10% ethanol (EtOH) as eluent (flow rate: 1.4 mL/min). Spectrophotometric detection at 210 nm (UV) was used to monitor the elution. A multistep desalting process was necessary for HA fractions. They were first diluted 1/6 in water and subsequently passed through a QAE Sephadex A-25 column, by eluting with water to eliminate free NaCl. In a second step, HA fractions were dialysed using membranes with a 3500 Da cut-off value. This passage was followed by gel permeation chromatography on a HW 40S TSK column, using 10% ethanol (EtOH) as eluent (flow rate: 5 mL/min). Spectrophotometric detection at 210 nm (UV) was used to monitor the elution.

### 2.4. Depolymerisation with Heparin Lyases

Exhaustive digestion with heparinases I, II and III was performed as described [23]. Exhaustive digestion with heparinase I was performed following the procedure reported [24]. Digestion with heparinase II was performed at 25 °C for 48 h in a total volume of 190 µL, containing 20 µL of a 20 mg/mL sample solution in water, 25 µL heparinase II (0.5 IU/mL in 10 mM monobasic potassium phosphate pH 7.0) and 145 µL of sodium/calcium acetate pH 7 solution (containing 2 mM of calcium acetate and 0.1 mg/mL bovine serum albumin (BSA). At the end of the incubation time, sample solutions were heated at 100 °C for 2 min to inactivate the enzymes and filtered through a 0.22-µm filter prior to LC-MS analysis.

### 2.5. LC-MS Analysis

LC-MS analyses were run on LC system (Platin Blue, Knauer, Berlin, Germany) coupled to ESI/Q-TOF mass spectrometer equipped with an electrospray interface and a high-resolution time-of flight mass analyser (Impact II, Bruker Daltonics, Bremen, Germany). Sample solutions (5 μL) were injected on C18 Kinetex column (100 mm × 2.1 mm i.d., with 2.6 μm particles, with pre-column filter, Phenomenex, Aschaffenburg, Germany) and eluted using the mobile phases A (10 mM dibutylamine and 10 mM acetic acid in water) and B (10 mM dibutylamine and 10 mM acetic acid in methanol). The digestion solutions with heparinases mixture, heparinase I and heparinase II were run using the method and gradient elution previously described [23]. The release of glucosamine monomers following enzymatic treatment was assessed by normalising the area of each monomer to the total area of the major regular disaccharides detected in each heparin sample. In particular, the peak area of each monomer was estimated from the extracted ion chromatogram (EIC) corresponding to its specific *m*/*z* value and normalised to the total peak area of the major disaccharides (ΔU-A_NS6S_, ΔU_2S_-A_NS_, ΔU_2S_-A_NAc6S_ and ΔU_2S_-A_NS6S_), which collectively represented the vast majority of glycosaminoglycan-derived species detected in each sample despite differences in their relative composition. Normalised values were calculated as follows: (monomer peak area/total peak area of the major disaccharides) × 100.

### 2.6. NMR Spectroscopy

For NMR experiments, samples were prepared by dissolving 35 mg of heparin in 0.6 mL of deuterium oxide (D_2_O). NMR spectra were acquired on a Bruker Avance III 600 MHz spectrometer (Karlsruhe, Germany) equipped with a 5 mm TCI cryoprobe at 303 K. One-dimensional ^1^H NMR experiments were performed using the *zgcppr* pulse sequence (Bruker library, Karlsruhe, Germany) with the following acquisition parameters: 16 scans, four dummy scans, relaxation delay of 12 s, spectral width of 18 ppm and transmitter offset of 4.7 ppm. Following exponential multiplication with a line broadening factor of 0.3 Hz, spectra were Fourier transformed, phase corrected, baseline corrected and calibrated. ^1^H-^13^C HSQC experiments were performed as described by Mauri et al. [25]. Briefly, spectra were acquired using 20 scans, 32 dummy scans, a relaxation delay of 2.5 s, spectral widths of 8 ppm (F2) and 80 ppm (F1) and transmitter offsets of 4.7 ppm (F2) and 80 ppm (F1). The one-bond coupling constant (^1^J_C–H_) was set to 150 Hz. A total of 1024 data points were collected for each of 320 increments. The free induction decays (FIDs) were processed using zero-filling to 4096 points in F2 and 1024 points in F1, followed by QSINE window multiplication in both dimensions and Fourier transformation. ^1^H-^13^C HSQC spectra were integrated using the standard TopSpin routine according to the procedure previously described [24] to determine the mono- and disaccharide composition of the analysed heparin samples. In addition, the percentage of trisulfated glucosamine residues at the non-reducing end was quantified from the relative intensity of the corresponding C2/H2 cross-peak with respect to the total C2/H2 signal of trisulfated glucosamine residues. According to the qualification of HSQC method for quantitative composition of heparins previously reported, the A_NS3S6x_ cross-peak values calculated from C-2 have an intermediate precision CV of 3.2% [25].

## 3. Results and Discussion

### 3.1. Affinity Chromatography Separation of NA and HA Fractions

Three heparin samples of different animal source, OMH-1, BMH-1 and PMH-1 were fractionated by affinity chromatography on an AT-Sepharose column to separate heparin chains based on their affinity toward AT into NA and HA fractions. Each sample was analysed in duplicate, demonstrating good reproducibility. After the extensive desalting of the resulting fractions, the average percentage recovery of NA fractions with respect to the total (NA + HA) was as follows: 61.5% NA-OMH-1; 73.7% NA-BMH-1 and 59.6% NA-PMH-1.

### 3.2. LC-MS Analysis After Heparinase II Treatment: Identification of Natural NRE Trisaccharide Species

The parent heparins, together with their corresponding fractions, were investigated by LC-MS following depolymerisation with heparinase II, with the aim of preserving the 3-*O*-sulfated (3S)-containing heparin sequences. This enzyme does not cleave uronic acid residues following glucosamines sulfated at position 3, whereas these sites are typically targeted by heparinase I, providing that uronic acid units are sulfated at position 2, as previously reported [18,19].

**Figure 1:**
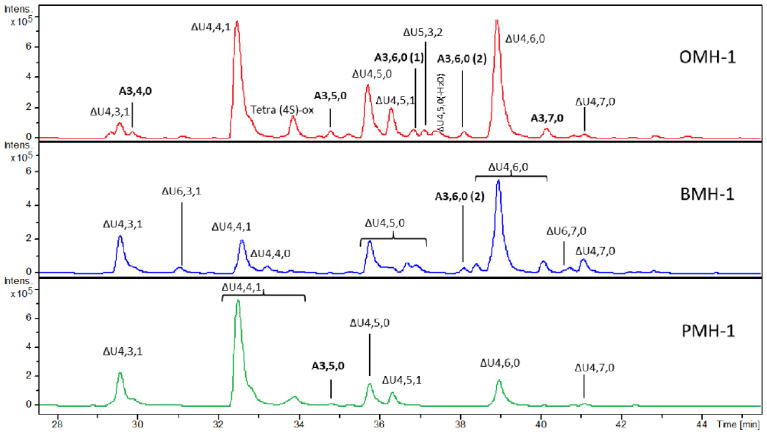
Liquid chromatography-mass spectrometry (LC-MS) chromatograms of parent heparins depolymerised with heparinase II: expanded view of the oligosaccharide region and the proposed structures of the main peaks. Trisaccharides are highlighted in bold.

The mass-to-charge ratio (*m*/*z*) data provided by mass spectrometry made it possible to calculate the true mass of the resulting ions enabling the determination of oligosaccharide composition and the proposal of structural hypotheses. For convenience, the oligosaccharide composition was described using a code composed of three numbers—representing the number of monosaccharide residues, sulfate groups and *N*-acetyl groups—preceded by a letter indicating the non-reducing end (NRE) residue. The symbol ΔU denotes that the first residue is a 4,5-unsaturated uronic acid (as expected from lyase activity), while the symbol A identifies oligomers that begin with a glucosamine residue (e.g., ΔU4,6,0 and A3,5,0, respectively, as reported in Figure 1).

Chromatographic profiles of the parent heparins displayed a series of oligomeric species in addition to disaccharides, varying in number and peak intensity according to the animal source. Complete LC-MS chromatograms are provided in the Supplementary Material (Figure S1), while the identified oligosaccharide species are shown in Figure 1. While only tri- and tetrasaccharides were detected in PMH-1, in BMH-1, hexasaccharides also appeared, as previously reported [20]. However, the OMH-1 profile exhibited the greatest diversity of oligosaccharide species, spanning tri- to pentasaccharides.

Since the heparinase II resistant tetrasaccharide component of heparin from ovine, bovine and porcine origin have already been studied [18], our attention was focused on the trisaccharides, a minor, yet significant component. The mass values of all the trimeric species detected correspond to sequences containing glucosamine units at both reducing end (RE) and NREs with differing numbers of sulfate groups, ranging from 4 to 7. Their sequence can be generically represented as A_NS3x6x_-I_2x_-A_NS3S6x_, where x indicates hydroxyl (OH) or sulfate substituent, and only in the case of A3,7,0 can the sequence be unambiguously identified as A_NS3S6S_-I_2S_-A_NS3S6S_. These highly sulfated, odd-numbered species, which appear to be particularly abundant in OMH-1 compared to BMH-1 and PMH-1, originate from the non-reducing end of heparin chains. Notably, they have not been detected previously using either the combined action of heparinases I-II-III or heparinase I alone.

**Figure 2:**
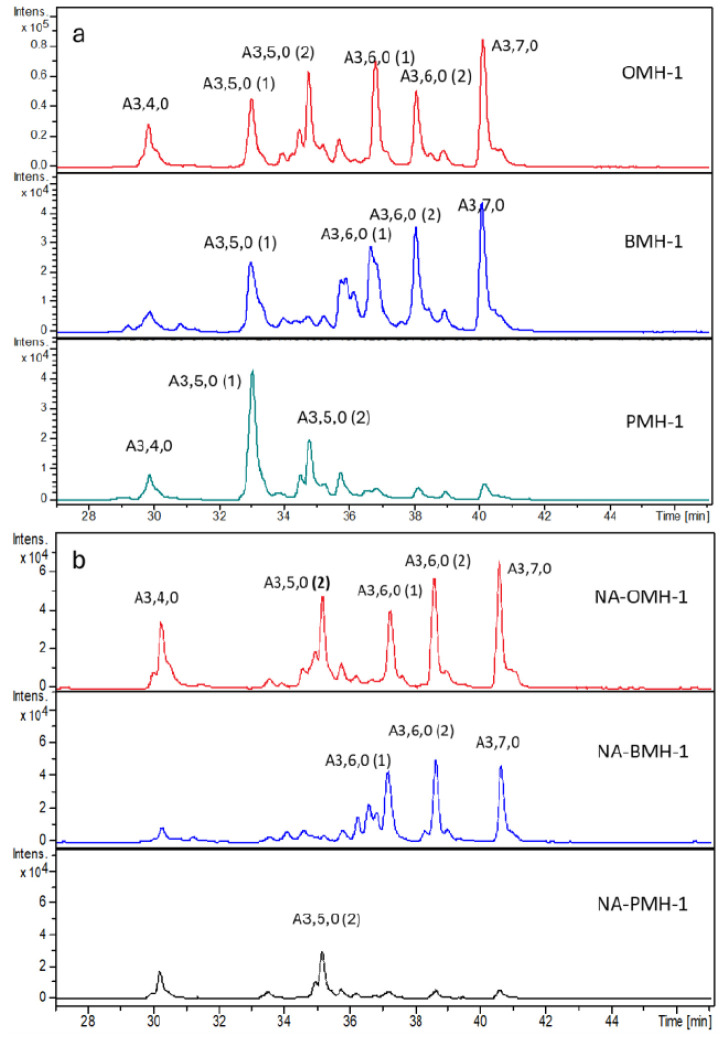

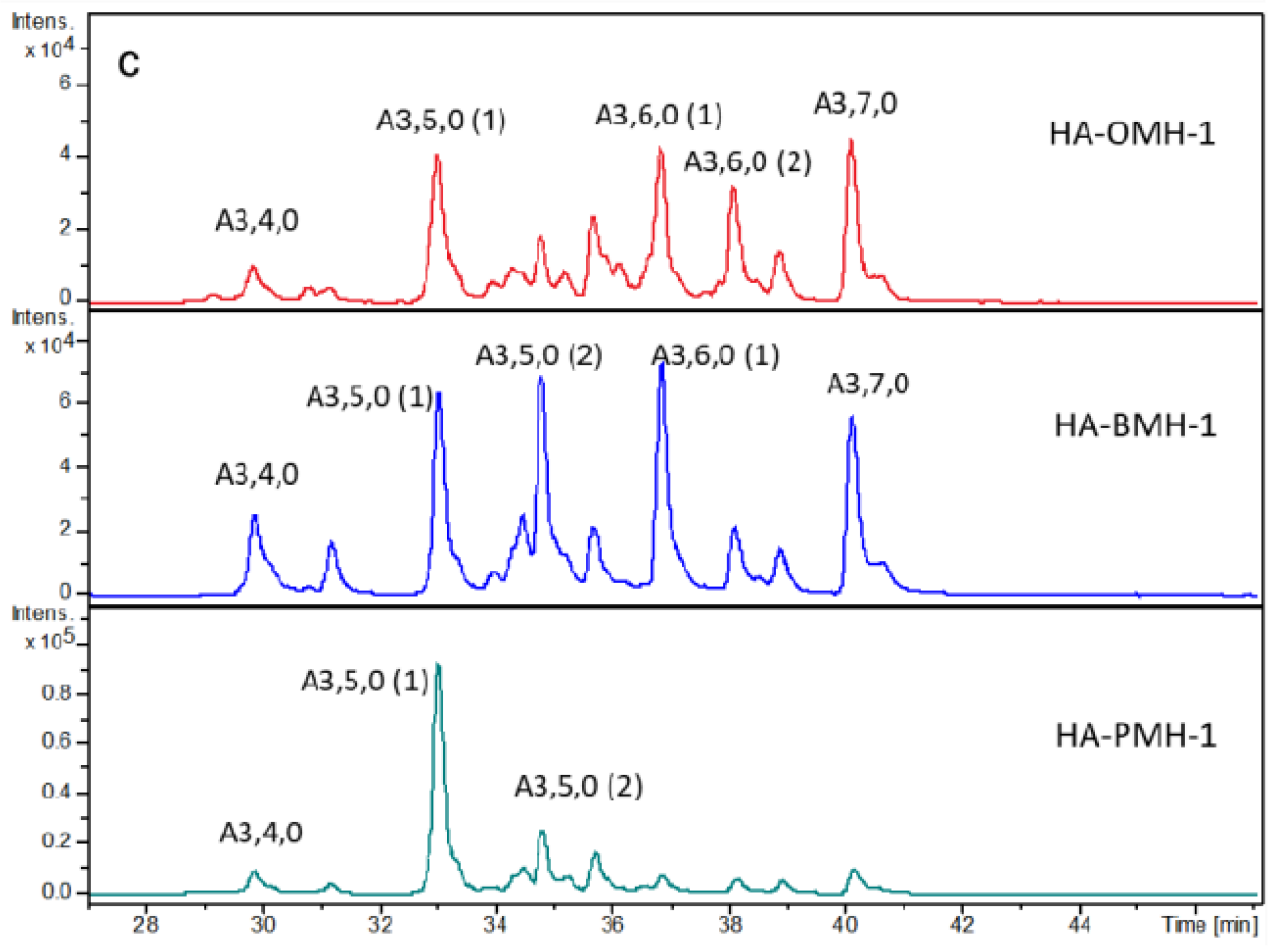
Reconstructed ion chromatograms (RICs) of the trisaccharide species generated by heparinase II digestion of (**a**) parent heparins (**b**) NA fractions and (**c**) HA fractions, with the proposed structure of the main peaks. The isomer number, corresponding to its elution order, is indicated in parentheses.

To better visualise the distribution of the trisaccharide species in the three parent heparins, the reconstructed ion chromatograms (RICs) of the selected peaks were generated and compared with those of the corresponding heparinase II digested NA and HA fractions (Figure 2). In Figure 2a, the RIC of OMH-1 exhibited two major isomers, (1) and (2), for A3,5,0 and A3,6,0 together with a single isomer both for A3,4,0 and, as expected, for A3,7,0. A similar composition was observed in BMH-1 profile, except for the reduced intensity of the peak from isomer A3,5,0 (2), whereas in PMH-1 profile, only the tetrasulfated and the prevailing pentasulfated species were observed, i.e., A3,4,0 and two isomers of A3,5,0, respectively. In Figure 2b, the NA heparin fractions of ovine, bovine and porcine origin are compared. At first glance, it appears clearly that the number of trisaccharide species has decreased, essentially due to the almost total absence of the isomer A3,5,0 (1) in all three NA fractions. The compositional profiles of the trisaccharide component of the HA fractions (Figure 2c) were similar to those of the corresponding parent heparins. Only a change in the relative intensity of some peaks was observed, such as a decrease of A3,5,0 (2) in both HA-OMH-1 and HA-PMH-1. Accordingly, the NRE sequence represented by the A3,5,0 (1) isomer appears to be exclusive to HA heparin chains, indicating that the structure of the immediately adjacent sequences is likely to be critical for interaction with AT.

**Figure 3:**
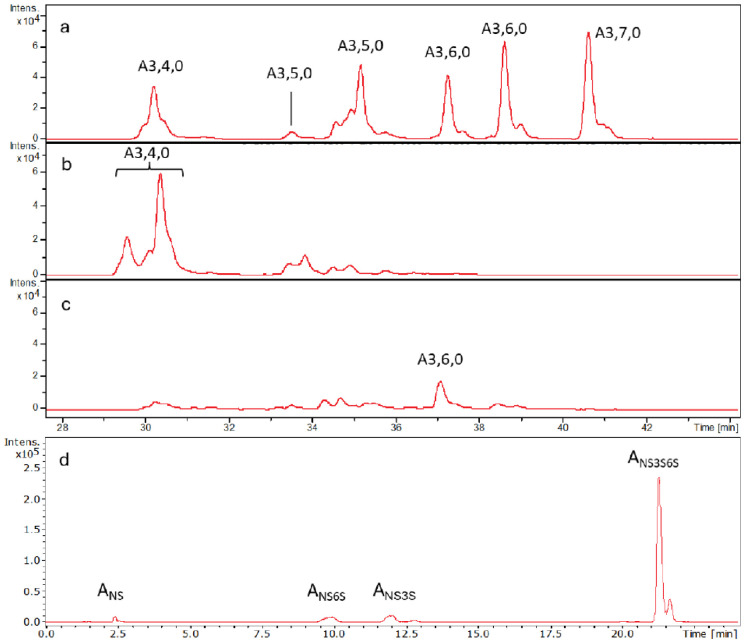
Reconstructed ion chromatograms (RICs) of (**a**–**c**) trisaccharide and (**d**) monosaccharide species derived from digestion of NA-OMH-1 with (**a**) heparinase II, (**b**) heparinase I, (**c**,**d**) heparinases I-II-III.

To investigate the possible sequences of the detected NRE trisaccharides, LC-MS was performed on the NA-OMH-1 fraction following depolymerisation with both heparinase I and the heparinase I-II-III cocktail. The corresponding RICs of the trisaccharide species were compared to that generated from heparinase II digestion (Figure 3).

Nearly all of the trisaccharide peaks disappeared upon heparinase I digestion, except for A3,4,0. Since heparinase I preferentially cleaves highly sulfated sequences, this result is consistent with the presence of a central, non-sulfated uronic acid, i.e., G/I_2OH_. Actually, heparinase I is unable to cleave the glycosidic linkage between A_NAc/NS_ (±6S) and G_2OH_ in the AT binding site [-A_NAc/NS6S_-G-A_NS3S6S_-I_2S_-A_NS6S_-]. The concerted action of the three heparinases I, II and III of the cocktail allowed the cleavage of that glycosidic bond, releasing all the NRE glucosamine monomers. This behaviour has already been reported by Mourier [26], whose chromatograms of OMH digested with the heparinase cocktail did not reveal any trisaccharide species. Indeed, heparinase III is known to exhibit broad substrate specificity, being capable of cleaving low-sulfated heparin regions containing both glucuronic and iduronic acid residues, with a preference—though not exclusivity—for *N*-acetylated sequences [27]. The residual presence of A3,6,0 species is likely to be caused by incomplete digestion by heparinase I, due to the lower enzyme-to-substrate ratio in the cocktail formulation compared with the single-enzyme condition.

### 3.3. LC-MS Analysis After Heparinase I-II-III Treatment: Appraisal of the Natural NRE Glucosamine Species

The remarkable prevalence of the trisulfated monomers A_NS3S6S_ detected in the NA-OMH-1 fraction, released after heparinase I-II-III digestion; in comparison with mono- and disulfated glucosamines led us to similarly evaluate the NRE glucosamine content, not only in the NA-BMH-1 and NA-PMH-1 fractions, but also in their parent heparins and in additional heparins of ovine, bovine and porcine origin. More precisely, two further UFH samples per animal source were considered.

The reversed phase-ion pair (RP-IP) chromatography method enabled the separation of four types of non-reducing end glucosamine residues: the monosulfated glucosamine A_NS_, the trisulfated glucosamine A_NS3S6S_ and two isomers of the disulfated glucosamine A_NS6S_ and A_NS3S_. The precise structure of these latter monomers was determined by MS/MS fragmentation in collision-induced-dissociation (CID) mode. The fragmentation spectrum of the first eluting isomer (RT 10 min) displayed characteristic ions attributable to A_NS6S_: the mass signal at *m*/*z* 137.99 corresponding to an *N*-sulfated glucosamine fragment, and additional signals at *m*/*z* 168.98 and *m*/*z* 198.99 indicative of 6-*O*-sulfation. Proceeding by exclusion, the second isomer (RT 12 min) was identified as A_NS3S_. This assignment was further supported by the absence of fragments typical of 6-*O*-sulfation and the appearance of a distinct signal at *m*/*z* 168.00, attributed to intra-ring cleavage involving the oxygen-C1 and C_3_-C_4_ bonds (Table 1).

An approximate assessment of the content of glucosamine monomers released from the non-reducing side of the heparin chains through complete enzymatic depolymerisation was achieved by normalising their individual areas to the total area of the major regular disaccharides detected in each heparin, as described in Materials and Methods.

**Figure 4:**
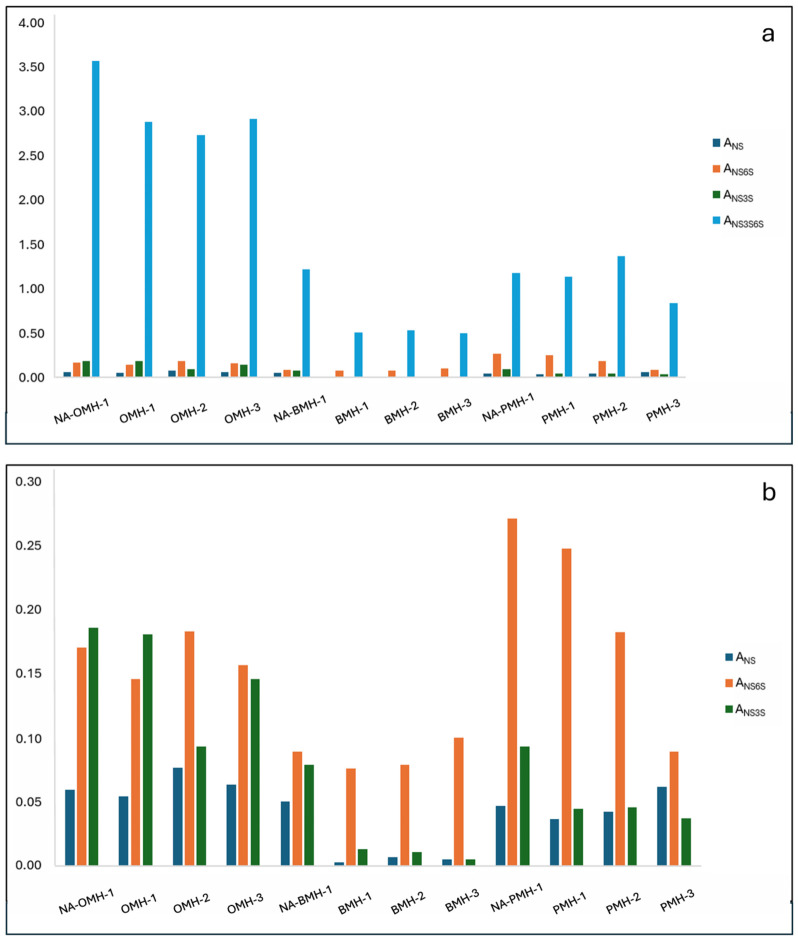
Relative quantification of glucosamine residues in ovine, bovine and porcine heparins, compared with the NA fraction of each animal source. Comparison of all the sulfated forms (**a**) and of mono- and di-sulfated species (**b**).

This normalisation was applied solely for comparative purposes to facilitate the identification of potential differences in monomer distribution patterns among samples. It was not intended to represent an absolute or relative quantitative measure of monomer abundance. The graphs in Figure 4 present a comparison of differently sulfated glucosamine residues in the three UFH samples analysed per animal source. Additionally, NA fractions of OMH-1, BMH-1 and PMH-1 are also compared. Although MS response is known to be influenced by several factors, particularly molecular structure and charge distribution, in this case the number of sulfate groups is the only variable, since only monosaccharide structures are being compared. Nevertheless, the predominance of the trisulfated glucosamine A_NS3S6S_ over the mono- and disulfated monomers remains evident across all heparin types, particularly in OMHs (Figure 4a). As regards the less sulfated forms of the glucosamine residue (Figure 4b), A_NS6S_ is the most prevalent in PMH, whereas the 3-*O*-sulfated disaccharide A_NS3S_ is more abundant in OMH than in BMH and PMH. Overall, these findings indicate a substantial presence of 3-*O*-sulfated glucosamines in OMHs. Interestingly, across all three heparin types, an enrichment of A_NS3S_ was observed particularly in the NA fractions.

The plausible non-reducing terminal trisaccharide sequences, compatible with the LC/MS results following digestion with heparinase II, heparinase I and heparinase I-II-III (Figure 3 and Figure 4), are reported in Table 2 in decreasing order of probability for OMH-1 and its NA fraction. However, the possible presence of 2-*O*-sulfated glucuronic acid (i.e., G_2S_ instead of I_2S_) in some of the penta-, hexa-, or heptasulfated species cannot be excluded, especially for porcine heparin [28]. Some of the detected NRE glucosamine residues may result from the endogenous activity of heparanase, an endo-β-d-glucuronidase that selectively cleaves heparan sulfate/heparin by hydrolysing the glycosidic bond between glucuronic acid and glucosamine residues. Nevertheless, the majority of the terminal 3-*O*-sulfated glucosamines, particularly in ovine heparin, are likely to be attributable to the biosynthetic process and may therefore represent a characteristic biosynthetic signature.

### 3.4. 2D-NMR Spectroscopy: Estimation of Natural NRE 3-O-Sulfated Glucosamine Content

To evaluate whether NRE 3-*O*-sulfated glucosamine can be quantified directly on intact samples—without requiring enzymatic depolymerisation—all UFHs and the isolated NA and HA fractions were examined using two-dimensional ^1^H/^13^C NMR. The integration of specific cross peaks in HSQC NMR spectra provided the mono/disaccharide compositional analysis of all the heparin samples. The determination of all the variously substituted glucosamine and uronic acid residues is presented in Table S1 (Supplementary Material). The compositional profiles here reported fall within the range of variability previously observed for heparins derived from the same animal sources [27].

**Figure 5:**
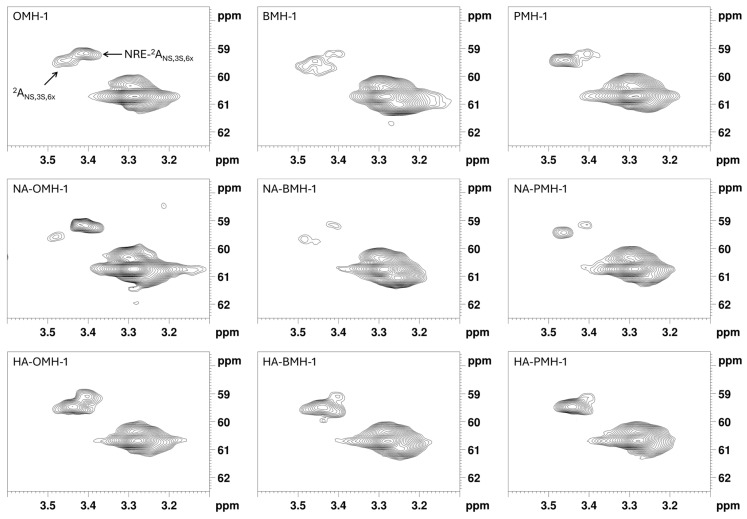
Heteronuclear single quantum coherence (HSQC) NMR spectra of parent ovine, bovine and porcine heparins and of their corresponding NA and HA fractions. Expansion of the H2/C2 *N*-sulfated-glucosamine regions. The cross peaks corresponding to the NRE and the intrachain ANS3S6x residues are indicated. (x = H or SO_3_^−^).

Figure 5 shows the H2/C2 *N*-sulfated glucosamine regions of the HSQC NMR spectra for all the analysed UFHs and their corresponding NA and HA fractions. In OMH-1, the cross peaks of the NRE and the intrachain A_NS3S6x_ residues are indicated [29]. The intensity of both cross-peaks varies in the different spectra of both the UFH and the derived fractions. As expected, the intrachain A_NS3S6x_ appears with the lowest intensity in all NA fractions and with the highest intensity in HA fractions. The cross-peak of NRE-A_NS3S6x_, however, is particularly evident in OMH as well as in its fractions, while it appears with reduced intensity in all other spectra. By integrating the observed signals, the percentage of the NRE-A_NS3S6x_ was calculated versus the overall 3-*O*-sulfated glucosamine content across all the UFHs and the isolated NA and HA fractions and compared with the percentage found within the AT-bs, represented by the G-(A_NS3S6x_) disaccharide (Table 3).

The total A_NS3S6x_ content varied between heparins of different sources, being relatively high in ovine heparins (6.7–8.1), slightly lower in porcine heparins (5.9–6.3) and much lower in bovine heparins (4.6–5.4). However, it is the percentage of NRE A_NS3S6x_ that most clearly discriminated heparins of different animal origin. In agreement with the indications provided by LC/MS analysis after enzymatic degradation with heparinases I, II and III, the highest content of NRE 3-*O*-sulfated glucosamine was found in ovine heparins (3.6–4.5%), whereas it was significantly lower in porcine heparins (1.8–2.4%) and lowest in bovine heparins (1.3–1.6%). Interestingly, the relatively high total A_NS3S6x_ content of NA-OMH-1 (5.4%) turned out to be located predominantly at the non-reducing terminal of heparin chain (4.3%), whereas in NA-PMH-1 and especially in NA-BMH-1, only a small portion of 3-*O*-sulfated residues were at the NRE (1.6% and 1.3%, respectively). This distinctive feature, which is therefore unrelated to anticoagulant activity, can be considered a structural determinant of ovine heparin, just as the NRE G-A_NS3S6S_ disaccharide characterises porcine heparin and the partial 2-*O*-sulfation of glucuronic acid residues is peculiar to bovine heparin [26,30].

This unexpectedly high abundance of 3-*O*-sulfated glucosamine located near the chain terminus also warrants consideration from a biological perspective. Recently, a library of 27 synthetic hexasaccharides, each containing the trisulfated glucosamine residue A_NS3S6S_, was evaluated to investigate the ligand requirements of several HS-binding proteins in addition to AT [31]. Interestingly, many of the proteins tested showed a preference for an I_2S_-A_NS3S6S_ disaccharide motif. However, in all of the synthetic hexasaccharides examined, this disaccharide sequence was positioned centrally within the oligosaccharide. It would therefore be valuable to investigate whether positioning the I_2S_-A_NS3S6S_ motif closer to the non-reducing end (NRE), as is unambiguously the case in the trisaccharide sequence A_NS3S6S_-I_2S_-A_NS3S6S_, preserves or enhances binding capacity.

Another interesting structural detail that emerges from Table 3 is the distribution of 3-*O*-sulfated residues in the HA fractions. As expected, in all HA fractions, the percentage of the total 3-*O*-sulfated glucosamine increased, due to the strong contribution of the component contained in the active site; this latter being 5.3%, 3.5% and 6.2% for OMH-1, BMH-1 and PMH-1, respectively. Interestingly, less than 50% of the total A_NS3S6x_ content of HA-BMH-1 was found to be located within the AT-bs and at the NRE, whereas for HA-OMH-1 and HA-PMH-1 the content was over 60%. A portion of the remaining A_NS3S6x_ sequences in HA-BMH-1, distributed along the heparin chain, is assumed to be incorporated into non-classical AT-binding sites, as reported for both heparan sulfate and heparin derivatives [32,33,34], probably containing 2-*O*-sulfated iduronic acid instead of glucuronic acid. This is in accordance with the finding of the heparinase II resistant tetrasaccharide ΔU4,7,0 compatible with the ΔU_2S_-A_NS6S_-I_2S_-A_NS3S6S_ sequence, the peak of which is particularly evident in BMH (Figure 1), as previously noted [19]. An octasaccharide containing the above sequence has been shown to bind and activate AT with an efficacy comparable to that of the classical AT-bs [35]. The presence of this highly sulfated sequence may account for the observation that the G-A_NS3S6x_ content in BMHs, despite being substantially lower than in OMHs and especially PMHs, does not correspond to a proportionally reduced anti-Xa activity [30].

## 4. Conclusions

The ongoing search for structural features relevant to understanding the properties of non-anticoagulant heparins, together with the growing need for more detailed characterisation of heparins derived from sources other than porcine tissue, led us to identify an unexpected structural peculiarity of ovine heparin. A remarkable high abundance of non-reducing 3-*O*-sulfated glucosamine residues, especially the trisulfated glucosamine A_NS3S6S_, was identified predominantly within highly sulfated NRE trisaccharide sequences and was preferentially associated with chains lacking AT affinity. This distinctive feature, which is therefore unrelated to anticoagulant activity, may be regarded as a structural marker of ovine heparin. Furthermore, the overall abundance of 3-*O*-sulfated glucosamine makes ovine mucosal heparin a valuable model for investigating the role of this key residue in a wide range of functional activities.

## Figures and Tables

**Table 1:** Mass spectrometry analysis of disulfated glucosamine isomers and CID-generated ion fragments.

*m*/*z*	z	Fragment-Generating Structure	Isomer 1 RT 10 min	Isomer 2 RT 12 min
137.9879	−1	*N*-sulfated glucosamine	x	x
167.9973	−1	3-*O*-sulfated glucosamine	-	x
168.9833	−1	6-*O*-sulfated glucosamine	x	-
198.9924	−1	6-*O*-sulfated glucosamine	x	-

**Table 2:** The possible sequences for NRE trisaccharides based on evidence from digestions with heparinase II, heparinase I and heparinase I-II-III.

NRE Trisaccharide Code	Sequence
A3,4,0	A_NS3S_-G/I-A_NS3S_ A_NS6S_-G/I-A_NS3S_ A_NS_-G/I-A_NS3S6S_
A3,5,0	A_NS3S_-I_2S_-A_NS3S_ A_NS6S_-I_2S_-A_NS3S_ A_NS_-I_2S_-A_NS3S6S_
A3,6,0	A_NS3S6S_-I_2S_-A_NS3S_ A_NS3S_-I_2S_-A_NS3S6S_ A_NS6S_-I_2S_-A_NS3S6S_
A3,7,0	A_NS3S6S_-I_2S_-A_NS3S6S_

**Table 3:** Percentage content of the total ANS3S6x and its NRE fraction, compared with the 3-O sulfated glucosamine included in the AT-bs.

	Total A_NS3S6x_	NRE A_NS3S6x_	G-(A_NS3S6x_)
OMH-1	7.8	4.2	2.0
NA-OMH-1	5.4	4.3	0.5 #
HA-OMH-1	13.3	3.2	5.3
OMH-2	6.7	3.6	1.5
OMH-3	8.1	4.5	1.8
BMH-1	5.4	1.5	1.0 #
NA-BMH-1	3.3	1.1	0.6 #
HA-BMH-1	10.4	1.3	3.5
BMH-2	4.7	1.6	1.0 #
BMH-3	4.6	1.3	0.9 #
PMH-1	5.9	1.9	3.1
NA-PMH-1	3.9	1.6	2.0
HA-PMH-1	10.4	0.7	6.2
PMH-2	6.3	2.4	2.6
PMH-3	5.9	1.8	3.0

Note: x = H or SO_3_^−^; # under the limit of quantification (Table S1).

## Data Availability

The original contributions presented in this study are included in the article/Supplementary Material. Further inquiries can be directed to the corresponding author.

## Supplementary Materials

The following supporting information can be downloaded at: https://www.mdpi.com/article/10.3390/biom16071025/s1, Figure S1. Full LC-MS chromatograms of parent heparins depolymerised with heparinase II. Table S1. Percent content of variously substituted glucosamine and uronic acid residues in different disaccharide sequences of parent ovine, bovine and porcine heparins and of derived NA and HA fractions [24].
