# Supplementary material for: The Content and Location of 3-O-Sulfated Glucosamine Enable Differentiation of Ovine, Bovine and Porcine Heparins
Source: Biomolecules. 2026 Jul 14;16(7):1025. doi: 10.3390/biom16071025 (PMC13406788; doi:10.3390/biom16071025)
Supplement: Supplementary file 1 [file biomolecules-16-01025-s001.zip › biomolecules-4377453-supplementary.pdf]

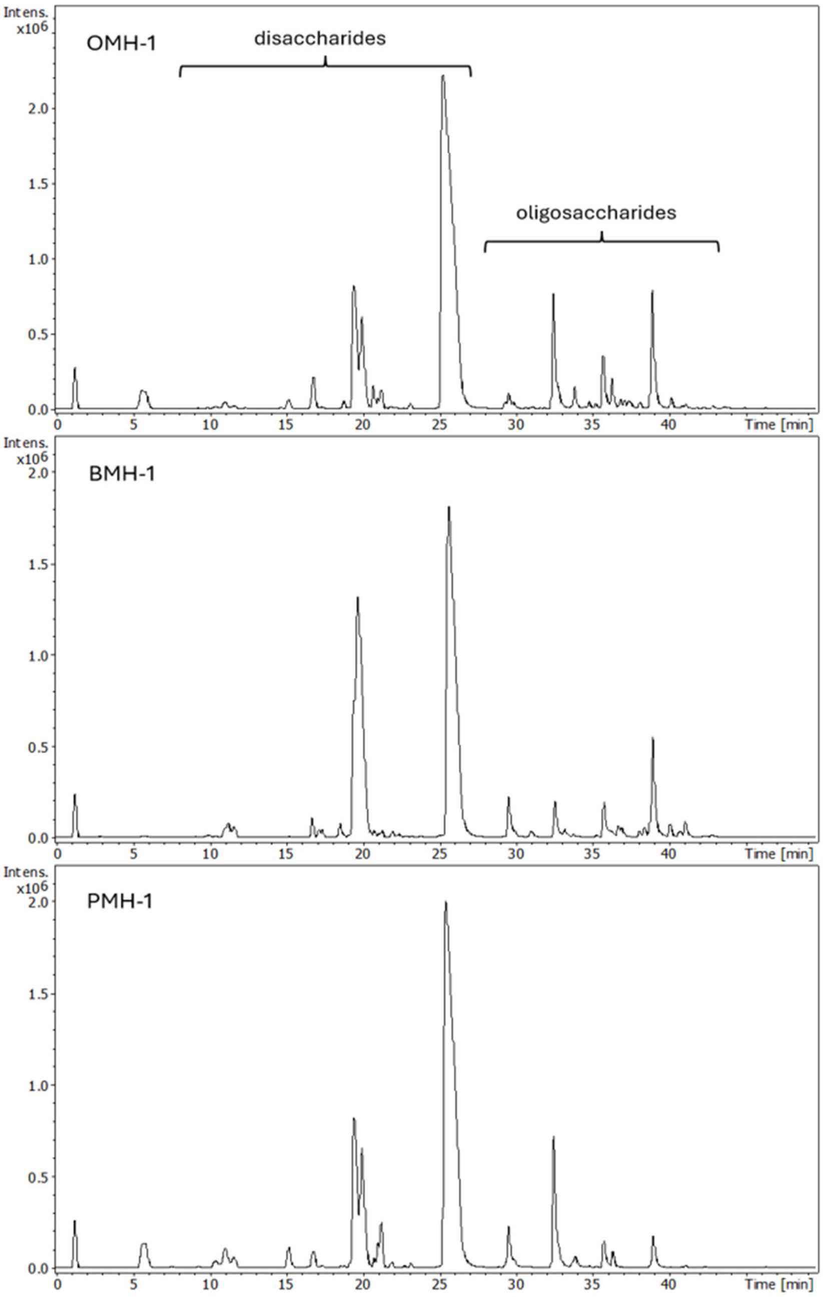

Figure S1. Full LC-MS chromatograms of parent heparins depolymerised with heparinase II.

Table S1. Percent content of variously substituted glucosamine and uronic acid residues in different disaccharide sequences of parent ovine, bovine and porcine heparins and of derived NA and HA fractions [24].

GLUCOSAMINE											
	ANS,6x-(G)	ANS,6x-(I)	ANS,6x-(I2S)	ANS,6x-(epox)	ANS,6x-(GalA)+Un.	ANS,3S,6x	ANAc,6x-(G)	ANAc,6x-(I)	ANH_2,6x	ANAc,6x αRed	ANS,6x αRed
OMH-1	12.0	4.8	66.8	<LOD	1.0 #	6.5	6.3	<LOD	1.3 #	<LOD	1.3 #
NA-OMH-1	9.8	5.5	72.5	<LOD	1.4	4.4	4.1	<LOD	<LOD	0.4 #	1.9
HA-OMH-1	15.4	8.3	55.5	<LOD	1.2 #	8.7	9.3	<LOD	0.9 #	<LOD	0.6 #
OMH-2	12.5	5.3	66.8	<LOD	1.4	4.9	6.8	<LOD	1.4	<LOD	0.9 #
OMH-3	9.8	5.3	68.1	0.35 #	0.7 #	7.2	5.1	<LOD	1.0	<LOD	2.4
BMH-1	15.4	4.5	67.7	<LOD	0.4 #	2.2	7.7	<LOD	1.1	0.5 #	0.5 #
NA-BMH-1	16.7	4.5	68.3	<LOD	1.0 #	1.0#	7.2	<LOD	<LOD	0.6 #	0.7 #
HA-BMH-1	17.2	6.7	58.1	<LOD	0.7 #	5.9	9.9	<LOD	0.8 #	<LOD	0.7 #
BMH-2	16.2	4.4	63.7	<LOD	4.54	2.1	6.8	<LOD	1.3 #	0.5 #	0.5 #
BMH-3	16.4	4.6	63.5	<LOD	4.70	2.1	6.2	<LOD	1.6 #	0.9 #	0.1 #
PMH-1	10.0	8.0	60.5	0.76 #	0.91 #	4.4	11.7	0.7 #	2.1	0.8 #	0.2 #
NA-PMH-1	11.4	7.1	62.2	0.81 #	1.36 #	3.0	10.3	0.8 #	2.0	0.9 #	0.1 #
HA-PMH-1	11.9	9.8	50.8	0.68 #	1.17 #	8.3	15.3	<LOD	1.6 #	0.6 #	<LOD
PMH-2	10.2	8.6	59.1	<LOD	2.26	5.2	11.9	0.6 #	1.9	<LOD	0.1 #
PMH-3	9.6	8.9	61.5	0.43 #	0.64 #	5.0	11.5	0.7 #	1.4	<LOD	0.3 #

URONIC ACIDS										
	G-(ANAc,6x)	G-(ANS,6x)	G-(ANS,3S,6x)	G2S	I-(ANy')	I-(ANy',6S)	I2S-(ANH_2,6x)	I2S-(ANy',3x,6x)	2,3-epoxide	GalA
OMH-1	2.5	5.5	2.0	0.9 #	1.3 #	4.91	2.1	80.3	<LOD	0.5
NA-OMH-1	2.2	5.5	0.5#	1.0 #	1.0 #	3.24	2.8	83.8	<LOD	<LOD
HA-OMH-1	3.8	8.1	5.3	<LOD	1.2 #	8.40	1.7 #	71.5	<LOD	<LOD
OMH-2	2.7	5.6	1.5	1.1 #	1.5	4.60	1.6	80.7	<LOD	0.7 #
OMH-3	1.9	5.5	1.8	0.8 #	0.8 #	4.57	1.7	82.6	0.4 #	<LOD
BMH-1	8.5	5.2	1.0#	1.9	3.2	1.33	1.3	77.5	<LOD	<LOD
NA-BMH-1	8.9	5.9	0.6#	2.4	3.2	1.14 #	1.4	76.4	<LOD	<LOD
HA-BMH-1	7.7	6.5	3.5	1.2 #	2.4	4.92	1.3 #	72.5	<LOD	<LOD
BMH-2	7.9	6.4	1.0#	2.0	3.1	1.75	2.1	73.7	<LOD	2.0
BMH-3	8.1	5.3	0.9#	1.8	3.3	1.75	1.5 #	74.6	<LOD	2.7
PMH-1	5.0	6.7	3.1	0.4 #	2.9	5.67	2.3	73.1	0.8 #	<LOD
NA-PMH-1	6.4	8.0	2.0	0.8 #	3.1	4.75	2.5	71.6	0.8 #	<LOD
HA-PMH-1	7.2	9.0	6.2	<LOD	3.1	9.08	1.5 #	63.1	0.8 #	<LOD
PMH-2	5.8	6.9	2.6	0.5 #	2.4	6.64	2.6	71.4	<LOD	1.1 #
PMH-3	6.1	6.5	3.0	0.4 #	2.3	5.29	1.7	74.3	0.4 #	<LOD

	LINKAGE REGION		DEGREE OF SULFATION	% 6S	LOD	LOQ
	LR	%ox Ser§				
OMH-1	2.8	L	2.65	83.3	0.42	1.42
NA-OMH-1	2.4	L	2.71	83.2	0.41	1.36
HA-OMH-1	3.6	L	2.59	87.3	0.53	1.77
OMH-2	3.2	L	2.63	82.8	0.42	1.39
OMH-3	1.3	M	2.70	83.9	0.30	1.01
BMH-1	2.5	H	2.24	50.9	0.30	1.01
NA-BMH-1	2.5	H	2.21	48.0	0.43	1.42
HA-BMH-1	2.9	L	2.34	63.9	0.50	1.66
BMH-2	2.6	H	2.22	50.9	0.48	1.60
BMH-3	2.1	H	2.22	50.3	0.51	1.71
PMH-1	4.6	L	2.41	76.2	0.37	1.25
NA-PMH-1	4.4	L	2.38	74.5	0.50	1.67
HA-PMH-1	4.5	L	2.34	78.6	0.51	1.71
PMH-2	4.4	H	2.42	77.0	0.44	1.46
PMH-3	2.6	H	2.46	78.7	0.43	1.42

# value lower than LOQ A = Glucosamine; G = Glucuronic acid; I = Iduronic acid;
GalA = Galacturonic acid; - X = H or SO_3^-; - Y = H or Ac or SO_3^-; - Y' = Ac or SO_3^-

§ Since it is impossible to quantify accurately the percentage of oxidized serine present in the linkage region, an approximate estimation is given
